# Effect of Nonintervention vs Oral Ibuprofen in Patent Ductus Arteriosus in Preterm Infants: A Randomized Clinical Trial

**DOI:** 10.1001/jamapediatrics.2020.1447

**Published:** 2020-06-15

**Authors:** Se In Sung, Myung Hee Lee, So Yoon Ahn, Yun Sil Chang, Won Soon Park

**Affiliations:** 1 Samsung Medical Center, Department of Pediatrics, Sungkyunkwan University School of Medicine, Seoul, Korea; 2 Statistics and Data Center, Samsung Medical Center, Sungkyunkwan University School of Medicine, Seoul, Korea

## Abstract

**Importance:**

Persistent patent ductus arteriosus (PDA) in preterm infants is associated with increased mortality and respiratory morbidities, including bronchopulmonary dysplasia (BPD). Despite recent increasing use of noninterventional approaches, no study to our knowledge has yet directly compared the nonintervention vs pharmacologic treatment for mediating PDA closure for decreasing mortality and preventing BPD.

**Objective:**

To determine the noninferiority of nonintervention vs oral ibuprofen treatment for PDA in decreasing BPD incidence or death in very preterm infants.

**Design, Setting, and Participants:**

A randomized, double-blind, placebo-controlled, noninferiority clinical trial was conducted on preterm infants (gestational age [GA] 23-30 weeks) with hemodynamically significant PDA (ductal size >1.5 mm plus respiratory support) diagnosed between postnatal days 6 and 14. Participants included 383 infants screened between July 24, 2014, and March 15, 2019.

**Interventions:**

Infants were stratified by GA and randomly assigned (1:1) to receive either oral ibuprofen (initial dose of 10 mg/kg followed by a 5-mg/kg dose after 24 hours and a second 5-mg/kg dose after 48 hours) or placebo.

**Main Outcomes and Measures:**

The primary outcome was BPD or death; the secondary outcomes included major morbidities and ductal closure rates. Per-protocol analysis was used.

**Results:**

Among 383 infants screened for participation, 146 infants were randomly assigned, with 72 in the nonintervention and 70 in the ibuprofen treatment group in the final analyses. The PDA closure rate at 1 week after randomization was significantly higher with ibuprofen (11 [34%]) than nonintervention (2 [7%]) in infants at GA 27 to 30 weeks (*P* = .007); however, the findings were not significant at GA 23 to 26 weeks (ibuprofen, 3 [8%] vs nonintervention, 1 [2%],* P* = .34). In addition, the ductal closure rates before hospital discharge (ibuprofen, 62 [89%] vs nonintervention, 59 [82%], *P* = .27) and device closure (ibuprofen, 2 [3%] vs nonintervention, 4 [6%], *P* = .40) were not significantly different between the 2 groups. The nonintervention approach was noninferior to ibuprofen treatment in terms of BPD incidence or death (nonintervention, 44%; ibuprofen, 50%; 95% CI, −0.11 to 0.22; noninferiority margin −0.2; *P* = .51). One infant in the ibuprofen arm received oral ibuprofen backup rescue treatment owing to cardiopulmonary compromise refractory to conservative management, and another infant in the ibuprofen group received surgical ligation; none of the infants in the placebo group received backup treatment.

**Conclusions and Relevance:**

Nonintervention showed noninferiority compared with ibuprofen treatment in closing of hemodynamically significant PDA and reduction of BPD or death. The noninferiority of nonintervention over ibuprofen might be attributable to the low efficacy of oral ibuprofen for closing PDA, especially in infants born at 23 to 26 weeks’ gestation.

**Trial Registration:**

ClinicalTrials.gov Identifier: NCT02128191

## Key Points

**Question:**

Is the nonintervention approach noninferior to oral ibuprofen treatment for hemodynamically significant patent ductus arteriosus with ductal size greater than 1.5 mm plus assisted respiratory support in very preterm infants?

**Findings:**

In a double-blind, randomized, placebo-controlled noninferiority clinical trial, 142 preterm infants at 23 to 30 weeks of gestation were enrolled after the first postnatal week between 2014 and 2018. On analysis of nonintervention vs oral ibuprofen, the incidence rates of bronchopulmonary dysplasia or death and other neonatal morbidities were not significantly different between the 2 study groups.

**Meaning:**

The nonintervention approach was noninferior to oral ibuprofen treatment in reducing bronchopulmonary dysplasia or death, probably owing to the low therapeutic efficacy of ibuprofen for closing patent ductus arteriosus in very preterm infants with hemodynamically significant patent ductus arteriosus.

## Teaser

This randomized clinical trial evaluates the noninferiority of nonintervention compared with oral ibuprofen therapy for reduction of bronchopulmonary dysplasia and death in very preterm infants with patent ductus arteriosus.

## Introduction

Persistent patent ductus arteriosus (PDA) in preterm infants is associated with increased mortality and worsened respiratory outcomes, including bronchopulmonary dysplasia (BPD)^1,2,3^; however, data supporting the causal association between PDA and these outcomes are lacking.^1,4,5^ Although nonsteroidal anti-inflammatory drugs (NSAIDs) are known to accelerate PDA closure, they have remained ineffective in improving mortality, long-term respiratory outcomes, or neurodevelopment, irrespective of the NSAIDs used.^1,4,5,6,7,8,9,10,11,12,13,14,15,16,17,18^ Multicenter cohort studies have reported substantial decreases in the rates of medical and surgical treatments of PDA in very low-birth-weight infants.^19,20,21,22^ However, it is unclear whether the conservative nonintervention approaches for PDA are associated with reduced BPD or death in preterm infants.^11,14,19,20,21,23,24,25,26,27^

In the recently conducted PDA—To Leave It Alone or Respond and Treat Early (PDA-TOLERATE) randomized clinical trial,^4^ early, routine pharmacologic treatment did not improve respiratory outcomes compared with conservative management. However, this study could have had a selection bias owing to the low enrollment rate of eligible infants, and the high rate of backup medical and surgical treatment of PDA might be another limitation. Therefore, in the present double-blind, randomized, placebo-controlled noninferiority trial, we compared the therapeutic efficacy of exclusive pharmacologic treatment with oral ibuprofen vs nonintervention placebo therapy, with few backup treatments for closing PDA and thereby reducing BPD or death in very preterm infants.

## Methods

### Study Design and Patients

This study was a single-center, randomized, double-blind, placebo-controlled, noninferiority, prospective clinical trial that aimed to verify the noninferiority of a nonintervention approach over exclusive pharmacologic treatment with oral ibuprofen. The study was conducted between July 24, 2014, and March 15, 2019, at the neonatal intensive care unit of Samsung Medical Center, Seoul, Korea. The study protocol was approved by the institutional review board of Samsung Medical Center, and written informed consent was obtained from both parents of each patient. This study followed the Consolidated Standards of Reporting Trials (CONSORT) reporting guideline for randomized clinical trials. The trial protocol and sample size calculation are available in Supplement 1.

The eligible patients were Korean preterm infants born and admitted to the Samsung Medical Center neonatal intensive care unit at gestational age (GA) 23 to 30 weeks. The infants required respiratory support, including high-flow nasal cannula, nasal continuous positive airway pressure, or mechanical ventilation, with hemodynamically significant PDA diagnosed at postnatal days 6 to 14. Hemodynamically significant PDA was defined as ductal size greater than 1.5 mm by an initial 2-dimensional echocardiogram (Acuson Sequoia C512; Siemens Medical Solutions) with predominant left to right shunt flow, using gain-optimized color Doppler, through PDA performed during postnatal days 6 and 14. The exclusion criteria were congenital heart disease, life-threatening congenital anomalies, predominant right to left shunt through PDA, severe intraventricular hemorrhage (grade ≥III), and contraindications to oral ibuprofen treatment, including life-threatening infection, bleeding tendency, thrombocytopenia with platelet count less than 50 × 10^3^/μL (to convert to ×10^9^/μL, multiply by 1), serum creatinine level greater than 2.0 mg/dL (to convert to millimoles per liter, multiply by 88.4), and necrotizing enterocolitis stage IIb or greater according to modified Bell criteria.^28^

### Sample Size

Based on a previous retrospective study,^11^ we estimated the proportion of infants developing BPD or death—the primary outcome of this study—in which the incidence rates of BPD or death were 55% in the period of mandatory PDA closure and 35% in the era of nonintervention, respectively. Using a 1-sided α level of .025, a sample size of 142 patients was required to detect a noninferiority margin of 20% with 80% power. The noninferiority margin of 20% was defined on the basis of the lower limit of the 95% CIs (19.8-20.2) of the difference in BPD incidence or mortality between the nonintervention group (18/51 [35%]) and treatment group (49/89 [55%]) in previous data.^11^ Because developing BPD or death could be affected not only by PDA management but also by diverse and complex host factors and variations in clinical practice, we assumed the margin of 20% to be clinically acceptable.

### Randomization and Blinding

This trial used a blocked randomization method stratified according to GA (23 weeks + 0 days to 26 weeks + 6 days, and 27 weeks + 0 days to 30 weeks + 6 days). The nonintervention and oral ibuprofen treatment groups were assigned at the same ratio (1:1) in each block. The randomization code table was prepared using SAS, version 9.4 (SAS Institute Inc). For the treatment arm, oral ibuprofen was administered via gavage tube within 24 hours after randomization was completed. The initial dose of 10 mg/kg was followed by a 5-mg/kg dose after 24 hours and a second 5-mg/kg dose after 48 hours. Normal saline with the same volume and schedule as used with ibuprofen was given as a placebo. The parents, hospital and research staff, and medical personnel directly involved in patient care were all blinded to group allocation throughout the study. Ibuprofen or placebo was administered by an unblinded medical professional who was not involved in patient treatment, and the administration was monitored by the other medical staff unaffiliated with the neonatal intensive care unit. Manual regurgitation of gastric residue was prohibited for at least 1 hour after the medication was administered to prevent the medical staff from knowing whether ibuprofen or placebo had been administrated.

### Concomitant Treatment

Judicious fluid restriction with diuretics administered as needed was maintained for the first 2 months of life.^11,29,30^ Fluid intake was decided based on body weight, serum sodium concentration and osmolality, and urine volume, and the target fluid volume was individualized for each infant after clinical evaluation of volume overload, including body weight change, hyponatremia, or cardiomegaly.

The need for backup rescue treatment with an additional cycle of ibuprofen or surgical ligation was decided by the attending neonatologist when the conditions of preterm infants with hemodynamically significant PDA deteriorated clinically with symptoms and signs of cardiopulmonary compromise that could not be explained otherwise. Backup rescue treatment was also considered when the condition was refractory to conservative management, including fluid restriction, use of diuretics and inotropic drugs, and modest increase in mean and end-expiratory ventilator airway pressure to reduce pulmonary blood flow.

### Clinical Course and Outcomes

Demographic information, biological data, and clinical outcomes, including death, were prospectively collected in this study. Clinical characteristics, including GA, birth weight, Apgar scores determined at 1 and 5 minutes, sex, small for GA status, mode of delivery, antenatal corticosteroid use, and chorioamnionitis, were analyzed. Ductal patency was monitored by follow-up serial echocardiographic scanning that was repeated regularly at 1, 2, and 4 weeks after randomization, at 36 weeks of GA, and at or after discharge until PDA closure.

Primary outcome measures included BPD incidence or death, where BPD was defined as the need for supplemental oxygen and/or positive pressure to maintain oxygen saturation greater than 90% at GA 36 weeks. Secondary outcome measures included BPD incidence, death before discharge, severe intraventricular hemorrhage (grade ≥III), retinopathy of prematurity (stage ≥3), necrotizing enterocolitis (stage ≥IIb), gastrointestinal surgery, and nosocomial sepsis confirmed on blood culture results.

### Statistical Analysis

We performed per-protocol analysis instead of intention-to-treat analysis; therefore, only infants who completed the intervention (all 3 doses of oral ibuprofen or normal saline) were included in our analysis. According to CONSORT reporting guideline, the per-protocol population is considered a conservative choice of analysis in noninferiority trials excluding superiority assumption.^31^ Baseline characteristics and outcomes were compared between the oral ibuprofen and nonintervention groups using a χ^2^ or Fisher exact test for categorical variables; a 2-tailed, unpaired *t*-test for parametric continuous variables; and the Wilcoxon rank sum test for nonparametric continuous variables, both in overall cohort and subgroup comparisons. The nonintervention approach was considered noninferior to ibuprofen treatment when the lower limit of the 95% CI for the difference in BPD incidence or death between the 2 groups was greater than −0.2, which was the prespecified margin of noninferiority. Binary logistic regression was used to calculate the adjusted odds ratio of ibuprofen vs nonintervention for the primary and secondary outcomes. We also performed a GA subgroup (GA 23-26 and GA 27-30 weeks) analysis to examine whether GA modified the effect of nonintervention vs oral ibuprofen on primary and secondary outcomes. The cumulative PDA closure rates were analyzed using Kaplan-Meier estimation, and the differences between both groups were analyzed by the log-rank test. Statistical analyses were performed using SAS, version 9.4 (SAS Institute Inc); R, version 3.0.3 (R Project for Statistical Computing); and Stata, version 14.0 (StataCorp LP). A *P* value <.05 was considered statistically significant.

## Results

### Study Population

**Figure 1.**
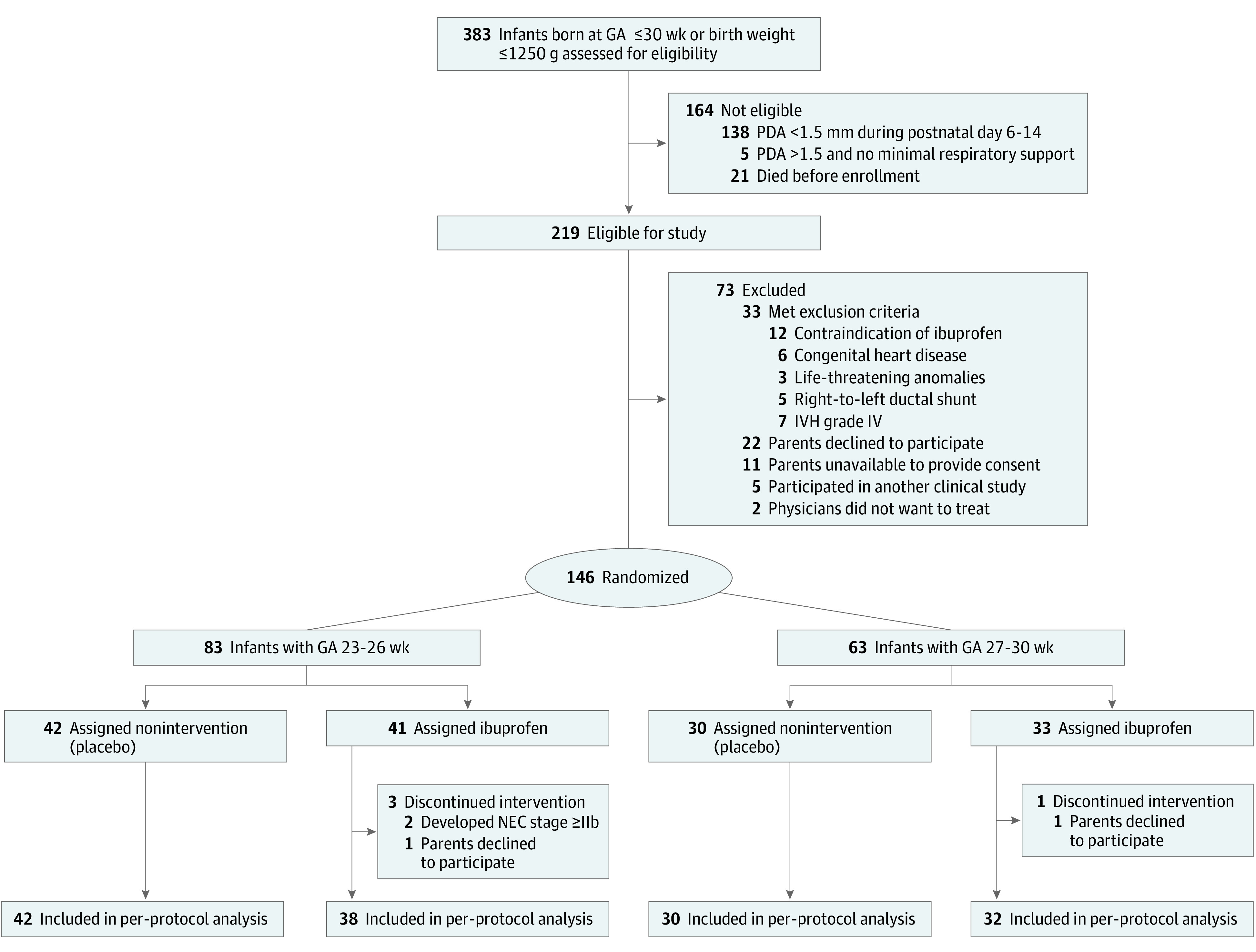
CONSORT Diagram GA indicates gestational age; IVH, intraventricular hemorrhage; NEC, necrotizing enterocolitis; and PDA, patent ductus arteriosus.

Of the 383 infants born at 23 to 30 weeks of gestation who were delivered at Samsung Medical Center and admitted to the neonatal intensive care unit during the study period, 146 infants were enrolled. The infants were stratified according to GA subgroups (GA 23-26 weeks, n = 83; GA 27-30 weeks, n = 63) and randomly allocated to the nonintervention and ibuprofen arms at a 1:1 ratio (nonintervention: 42 and 41 infants in GA 23-26 weeks group; ibuprofen: 30 and 33 infants in GA 27-30 weeks group) (Figure 1). Because we performed the per-protocol analysis, 4 infants in the oral ibuprofen group were excluded owing to incompletion of the ibuprofen cycle and 142 infants were included in the final analyses.

Demographic and clinical characteristics of each study group are presented in Table 1. The nonintervention group comprised significantly more male infants (41 [57%]) than the oral ibuprofen group (28 [40%]) (*P* = .04); antenatal corticosteroid use, cesarean delivery, and multiple births were not significantly different between the study groups.

**Table 1.** Clinical Characteristics of the Trial Population

Characteristic	Total (n = 142)		Gestational age			
			23-26 wk (n = 80)		27-30 wk (n = 62)	
	Nonintervention (n = 72)	Ibuprofen (n = 70)	Nonintervention (n = 42)	Ibuprofen (n = 38)	Nonintervention (n = 30)	Ibuprofen (n = 32)
**Prenatal or neonatal variables**						
Gestational age, mean (SD), wk	26.7 (2.0)	26.8 (2.1)	25.3 (1.0)	25.3 (1.1)	28.7 (1.3)	28.7 (1.3)
Birth weight, mean (SD), g	915 (243)	893 (256)	785 (132)	740 (145)	1097 (248)	1086 (243)
Male sex, No. (%)	41 (57)	28 (40)	24 (57)	16 (42)	17 (57)	12 (38)
Apgar score, mean (SD)						
At 1 min	5.3 (1.7)	5.2 (1.9)	4.9 (1.5)	4.3 (1.5)	5.7 (1.9)	6.2 (1.9)
At 5 min	7.6 (1.3)	7.5 (1.4)	7.3 (1.3)	6.9 (1.2)	7.9 (1.2)	8.1 (1.3)
Multiple births, No. (%)	23 (32)	22 (31)	14 (33)	14 (37)	9 (30)	8 (25)
Premature rupture of membranes (≥24 h), No. (%)	17 (24)	11 (16)	15 (36)	8 (21)	2 (7)	3 (9)
Pregnancy-induced hypertension, No. (%)	6 (8)	12 (17)	0	2 (5)	6 (20)	10 (31)
Chorioamnionitis, No. (%)	43 (60)	37 (53)	31 (74)	29 (76)	12 (40)	8 (25)
Antenatal corticosteroid use, No. (%)	68 (94)	65 (93)	40 (95)	36 (95)	28 (93)	29 (91)
Cesarean delivery, No. (%)	54 (75)	56 (80)	26 (62)	27 (71)	28 (93)	29 (91)
Small for gestational age, No. (%)	12 (17)	14 (20)	4 (10)	5 (13)	8 (27)	9 (28)
Intubation at 24 h, No. (%)	64 (89)	61 (87)	42 (100)	36 (95)	22 (73)	25 (78)
At randomization, No. (%)						
Intubation	40 (56)	37 (53)	31 (74)	26 (68)	9 (30)	11 (34)
N-CPAP	18 (25)	18 (26)	10 (24)	10 (26)	15 (50)	16 (50)
HFNC	14 (19)	15 (21)	1 (2)	2 (5)	6 (20)	5 (16)
Inotropes before randomization	7 (10)	7 (10)	5 (12)	5 (13)	2 (7)	2 (6)
**PDA-associated variables**						
PDA size at randomization, mean (SD)	2.5 (0.6)	2.5 (0.5)	2.4 (0.5)	2.4 (0.5)	2.8 (0.6)	2.6 (0.6)
LA/Ao ratio, mean (SD)	1.69 (0.35)	1.61 (0.41)	1.65 (0.34)	1.57 (0.37)	1.67 (0.47)	1.74 (0.38)
E/A ratio, mean (SD)	0.88 (0.21)	0.85 (0.20)	0.84 (0.22)	0.78 (0.19)	0.93 (0.19)	0.95 (0.17)
NT-proBNP at randomization, mean (SD), pg/mL	14 514 (10 363)	13 355 (10 126)	13 848 (9922)	15 167 (10 707)	15 447 (11 056)	11 203 (9088)
Age at ibuprofen or placebo administration, mean (SD), d	8.4 (2.5)	8.3 (2.3)	8.6 (2.2)	8.9 (2.3)	8.3 (2.8)	7.5 (2.4)

Abbreviations: E/A, early to late transmitral peak flow velocity; HFNC, high-flow nasal cannula; LA/Ao, left atrium/aorta; N-CPAP, nasal continuous positive airway pressure; NT-proBNP, N-terminal probrain natriuretic peptide; PDA, patent ductus arteriosus.

### PDA Status

Regarding PDA-associated variables at enrollment, there were no significant differences in the PDA size, left atrium-to-aorta ratio, early-to-late transmitral inflow velocities ratio, N-terminal probrain natriuretic peptide levels measured as a biomarker for hemodynamically significant PDA, and age at oral ibuprofen or placebo administration between the study groups (Table 1).

**Figure 2.**
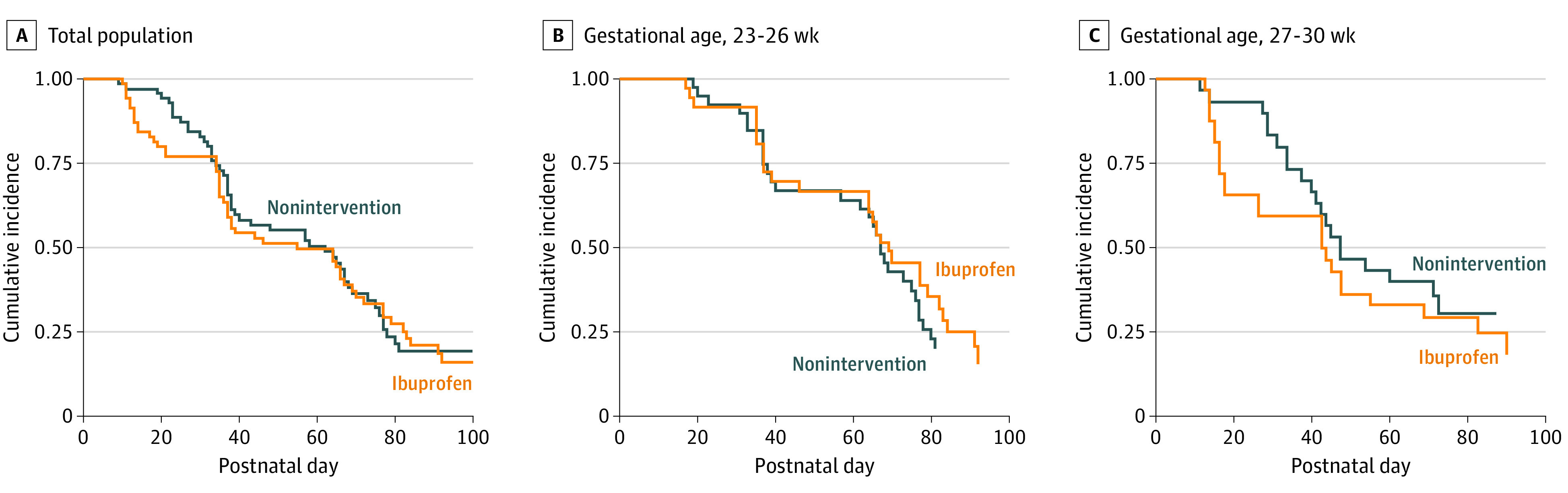
Cumulative Incidence of Ductal Patency Over Postnatal Day Between Nonintervention and Ibuprofen Groups A, Total population; B, Infants with gestational age 23 to 26 weeks; C, Infants with gestational age 27 to 30 weeks.

Regarding the hemodynamically significant PDA outcome, although the ductal closure rate at 1 week after randomization was significantly higher in the oral ibuprofen group (11 [34%]) than in the nonintervention group (2 [7%]) among infants with GA 27 to 30 weeks (*P* = .007), this result was not observed for those with GA 23 to 26 weeks; 62 infants (89%) in the oral ibuprofen group and 59 infants (82%) in the nonintervention groups underwent PDA closure before hospital discharge (*P* = .27). In addition, the follow-up ductal closure rate (ibuprofen, 2 [3%] vs nonintervention, 4 [6%], *P* = .40) and the ultimate failure rate of ductal closure requiring transcatheter PDA occlusion were not significantly different between the study groups (Table 2, Figure 2).

**Table 2.** Primary and Secondary Outcomes

Outcome	Total (n = 142)			Gestational age					
				23-26 wk (n = 80)			27-30 wk (n = 62)		
	Nonintervention (n = 72)	Ibuprofen (n = 70)	*P* value	Nonintervention (n = 42)	Ibuprofen (n = 38)	*P* value	Nonintervention (n = 30)	Ibuprofen (n = 32)	*P* value
**Primary outcome**									
BPD or death	32 (44)	35 (50)	.51	26 (62)	25 (66)	.72	6 (20)	10 (31)	.31
**Secondary outcomes**									
Major morbidity/mortality, No. (%)									
BPD	27/67 (40)	29/64 (45)	.56	21/37 (57)	21/34 (62)	.67	6 (20)	8/30 (27)	.54
Death before discharge	6 (8)	6 (9)	.96	6 (14)	4 (11)	.74	0	2 (6)	.49
IVH (grade ≥III)	4 (6)	2 (3)	.68	3 (7)	1 (3)	.62	1 (3)	1 (3)	>.99
ROP (stage ≥3)	14 (19)	15 (21)	.77	13 (31)	14 (37)	.58	1 (3)	1 (3)	>.99
NEC (stage ≥IIb)	3 (4)	7 (10)	.21	3 (7)	4 (11)	.70	0	3 (9)	.12
Gastrointestinal surgery	6 (8)	9 (13)	.38	6 (14)	5 (13)	.88	0	4 (13)	.11
Sepsis	4 (6)	10 (14)	.08	4 (10)	9 (24)	.09	0	1 (3)	.49
Oxygen or ventilator dependency until hospital discharge, (IQR), d									
Ventilator support	19 (2-31)	17 (1-36)	.76	25 (18-39)	28 (6-48)	.87	2 (0-18)	3 (0-18)	.66
N-CPAP/HFNC	58 (28-72)	51 (26-73)	.60	71 (66-75)	73 (51-80)	.90	26 (12-46)	27 (9-45)	.70
Supplemental oxygen	22 (8-41)	24 (6-34)	.54	36 (17-43)	30 (24-40)	.91	18 (3-26)	8.5 (1-23)	.25
PDA-related outcomes, No. (%)									
Surgical ligation	0	1 (1)		0	1 (3)		0	0	
Backup oral ibuprofen treatment	0	1 (1)		0	0		0	1 (3)	
NT-proBNP 2 wk after randomization, mean (SD), pg/mL	13 812 (13 468)	11 552 (11 480)	.43	19 206 (13 384)	16 361 (11 040)	.50	6800 (10 028)	5813 (9249)	.53
Ductal closure, No. (%)									
1 wk After randomization	3 (4)	14 (20)	.003	1 (2)	3 (8)	.34	2 (7)	11 (34)	.007
At 36 wk PMA	50 (69)	51 (73)	.49	30 (71)	27 (71)	.86	20 (67)	24 (75)	.2
Before hospital discharge	59 (82)	62 (89)	.27	38 (90)	34 (89)	>.99	21 (70)	6 (81)	.12
Transcatheter PDA occlusion at OPD	4 (6)	2 (3)	.40	1 (2)	1 (3)	.97	3 (10)	1 (3)	.27
Other outcomes									
Full enteral feeding (>120 mL/kg/d), mean (SD), d	26.3 (14.4)	29.2 (16.3)	.39	32.0 (13.3)	36.1 (16.0)	.27	20.1 (13.1)	21.0 (12.6)	.78
Oliguric renal failure, No. (%)^a^	6 (8)	8 (11)	.54	6 (14)	7 (18)	.62	0	1 (3)	.33
Nonoliguric renal dysfunction, No. (%)^b^	9 (13)	15 (21)	.16	6 (14)	9 (24)	.28	2 (7)	7 (22)	.09
Highest serum creatinine after randomization, mean (SD), mg/dL	1.2 (0.9)	1.0 (0.4)	.95	1.5 (1.1)	1.2 (0.4)	.75	0.8 (0.4)	0.8 (0.3)	.38
Body weight at 36 wk PMA, mean (SD), g	1949 (360)	1894 (418)	.42	1885 (312)	1815 (380)	.40	2028 (403)	1986 (447)	.71

Abbreviations: BPD, bronchopulmonary dysplasia; HFNC, high-flow nasal cannula; IQR, interquartile range; IVH, intraventricular hemorrhage; N-CPAP, nasal continuous positive airway pressure; NEC, necrotizing enterocolitis; NT-proBNP, N-terminal probrain natriuretic peptide; OPD, outpatient department; PDA, patent ductus arteriosus; PMA, postmenstrual age; ROP, retinopathy of prematurity.

SI conversion factor: To convert serum creatinine to millimoles per liter, multiply by 88.4.

^a^ Urine output less than 0.5 mL/kg/d for more than 24 hours combined with serum creatinine level greater than or equal to 2.0 mg/dL.

^b^ Serum creatinine level greater than or equal to 2.0 mg/dL without oliguria.

### Backup Rescue Treatment

In the oral ibuprofen arm, one infant received oral ibuprofen backup rescue treatment owing to cardiopulmonary compromise refractory to conservative management upon the decision of the attending neonatologist and the pediatric cardiologist (Table 2). Another infant in the treatment group received surgical ligation owing to high serum creatinine levels, which is a contraindication for use of ibuprofen. In the nonintervention arm, no infant received surgical ligation or oral ibuprofen as backup rescue treatment.

### Outcomes

There was no significant difference in the primary outcome of BPD or death between the study groups (nonintervention, 44% vs ibuprofen, 50%; risk difference, 6%; 95% CI, −0.11 to 0.22; noninferiority margin, −0.2; *P* = .51) (Table 2). The incidence rates of other adverse outcomes, including intraventricular hemorrhage (grade ≥III), retinopathy of prematurity (stage ≥3), and necrotizing enterocolitis (stage ≥IIb), were also not significantly different between the study groups. In multivariate analyses, although the adjusted odds ratios for ductal closure at 1 week after randomization in the oral ibuprofen group were significantly increased (8.77; 95% CI, 2.08-36.87), those for ductal closure before hospital discharge along with BPD incidence or death and other adverse outcomes were not significantly different between the study groups (Table 3).

**Table 3.** Multivariable Analysis

Variable	Ibuprofen over nonintervention, aOR (95% CI)^a^		
	Total (n = 142)	Gestational age	
		23-26 wk (n = 80)	27-30 wk (n = 62)^b^
BPD or death	1.61 (0.72-3.63)	1.25 (0.43-3.59)	2.34 (0.61-8.87)
BPD	1.48 (0.64-3.41)	1.25 (0.43-3.62)	1.94 (0.49-7.67)
Death before hospital discharge	1.14 (0.32-4.04)	0.70 (0.15-3.13)	NA
IVH (grade ≥III)	0.50 (0.08-3.25)	0.17 (0.01-4.19)	NA
ROP (stage ≥3)	1.00 (0.41-2.48)	1.08 (0.41-2.86)	0.94 (0.05-17.60)
NEC (stage ≥IIb)	2.52 (0.60-10.52)	1.46 (0.29-7.26)	NA
Gastrointestinal surgery	1.66 (0.54-5.14)	0.83 (0.22-3.17)	NA
Sepsis	3.60 (0.91-14.24)	3.19 (0.75-13.52)	NA
Ductal closure			
1 wk After randomization	8.77 (2.08-36.87)	4.14 (0.28-58.70)	11.07 (1.82-67.30)
Before 36 wk PMA	1.38 (0.59-3.21)	1.08 (0.32-3.66)	1.94 (0.55-6.82)
Before hospital discharge	1.90 (0.69-5.21)	1.07 (0.23-4.88)	3.12 (0.75-12.91)

Abbreviations: aOR, adjusted odds ratio; BPD, bronchopulmonary dysplasia; IVH, intraventricular hemorrhage; NA, not applicable; NEC, necrotizing enterocolitis; PMA, postmenstrual age; ROP, retinopathy of prematurity.

^a^ Odds ratio adjusted by gestational age, sex, Apgar score at 5 minutes, multiple birth, premature rupture of membranes (≥24 hours), and chorioamnionitis.

^b^ NA indicates that there were insufficient numbers to calculate the aORs.

## Discussion

We performed a double-blind, randomized, placebo-controlled trial to test the noninferiority of nonintervention over oral ibuprofen treatment in very preterm infants. We were unable to locate previous randomized studies comparing the therapeutic efficacy of exclusive pharmacologic treatment vs nonintervention with few backup medical or surgical treatments for closing PDA and reducing BPD or death. In the present study, while oral ibuprofen treatment significantly enhanced the ductal closure rate at 1 week after randomization compared with the nonintervention approach in the 27- to 30-week GA subgroup, but not in the 23- to 26-week GA subgroup, the nonintervention approach was noninferior to oral ibuprofen treatment in reducing the incidence of BPD or death in very preterm infants. However, the noninferiority of nonintervention over pharmacologic treatment might be attributable to the low efficacy of oral ibuprofen for closing PDA, especially in the infants with GA 23 to 26 weeks.

The failure rate of NSAID treatment for closing PDA has been reported to depend on GA.^32,33,34^ Van Overmeire et al^32^ showed that infants with GA less than or equal to 26 weeks had greater than 4.26-fold higher odds of treatment failure than those with GA 31 to 32 weeks. In a similar manner, our data showed accelerated ductal closure 1 week after oral ibuprofen treatment; however, the change was confined to infants at GA 27 to 30 weeks but not in infants with GA 23 to 26 weeks. As prolonged exposure to hemodynamically significant PDA for more than 7 to 13 days increases the incidence of BPD or death in very preterm infants,^35^ our results of nonsignificant reduction in BPD or death with oral ibuprofen compared with nonintervention might be attributable to the low therapeutic efficacy of oral ibuprofen for closing PDA, leading to no substantial difference in the duration of exposure to hemodynamically significant PDA between the groups. Although higher odds of PDA closure have been reported with oral ibuprofen than with intravenous ibuprofen or indomethacin,^36^ further studies using other NSAIDs are necessary to clarify whether there is a variation in the effectiveness of NSAIDs for closing PDA and thereby improving BPD or death.

However, our data showed that 89% and 82% PDA closure rates at hospital discharge with limited or no backup treatment in the oral ibuprofen and nonintervention groups, respectively, were relatively higher than the previously known overall PDA closure rate of 67% after NSAID treatment in preterm infants.^36^ In contrast to our trial, the PDA-TOLERATE randomized clinical trial showed the rates of ligation or persistent PDA at discharge as 32% and 39% in the early-rescue and conservative treatment groups, respectively.^4^ Furthermore, our data of 44% and 50% BPD or death rates in the nonintervention and oral ibuprofen groups, respectively, were lower than those in the PDA-TOLERATE trial (58% and 57% observed in the conservative and early-rescue treatment groups, respectively). Compared with the previous studies, our results suggest that most patients with hemodynamically significant PDA could eventually undergo spontaneous closure even with the nonintervention approach.

The primary outcome of this trial was the composite of BPD incidence or mortality. There might be a concern that death could alter the potential for BPD diagnosis. It is possible that meaningful changes could occur in these diagnoses without affecting the study’s composite outcome, for example, if the mortality increased in one treatment arm with the result that BPD was decreased. In this scenario, the outcome of BPD or mortality would show little change. Therefore, we also separately presented the BPD incidence and mortality as secondary outcomes and found that BPD incidence and mortality were not significantly different between the nonintervention and ibuprofen groups.

There is a lack of a clear definition of hemodynamically significant PDA shunt size and clinical illness severity requiring active treatment.^3,37,38,39^ In the present trial, the criterion for the enrollment of infants with hemodynamically significant PDA was defined as ductal size greater than 1.5 mm with respiratory failure requiring assisted respiratory support in very preterm infants.^4^ Determining the exact timing of PDA treatment is another issue that needs to be addressed for successful clinical translation. Owing to the spontaneous closure of PDA before the end of the first postnatal week in more than 30% to 40% of extremely preterm infants,^20,22,30^ previous randomized clinical trials that enrolled infants within the first few days of birth might be confounded by the high spontaneous closure rate of PDA, even in the conservative nonintervention arm of those studies.^20,22,30,40^ To avoid enrolling infants whose PDA might close spontaneously within a few days after enrollment,^4^ we waited until the end of the first week before enrolling the infants.

### Limitations

The limitations of the present study include its single-center design and a relatively large noninferiority margin that limited the study’s power to detect small differences in the efficacy of the treatments according to stratified GA subgroups, although the sample size was determined using appropriate calculations. Nevertheless, less variation in clinical management policies and the prospective, double-blind, randomized, placebo-controlled design of the trial might be strengths of this single-center study. Other limitations include the arbitrary definition of hemodynamically significant PDA defined only on the basis of the PDA size and respiratory assistance dependency and the lack of long-term neurodevelopmental outcome data.

## Conclusions

The nonintervention approach after the first postnatal week was noninferior to oral ibuprofen treatment for closing hemodynamically significant PDA, defined as PDA size greater than 1.5 mm with respiratory assistance dependency and, thereby, for reducing the incidence of BPD or death in very preterm infants with GA 23 to 30 weeks. The noninferiority of nonintervention over pharmacologic treatment in this single-center trial with a relatively small number of infants might be attributable to the low efficacy of oral ibuprofen for closing PDA, especially in infants with GA 23 to 26 weeks.

## Notes

Supplement 1. Trial Protocol and Sample Size Calculation

Supplement 2. Data Sharing Statement
