# Supplementary material for: Effect of Nonintervention vs Oral Ibuprofen in Patent Ductus Arteriosus in Preterm Infants: A Randomized Clinical Trial
Source: JAMA Pediatr. 2020 Jun 15;174(8):1–9. doi: 10.1001/jamapediatrics.2020.1447 (PMC7296457; doi:10.1001/jamapediatrics.2020.1447)
Supplement: Supplement 1. — Trial Protocol and Sample Size Calculation [file jamapediatr-174-755-s001.pdf]

Comparison of Efficacy and Safety between No Treatment and Ibuprofen Treatment for Patent Ductus Arteriosus in Extremely Low Gestational Age Newborns: A Randomized, Double-blind, Placebo-controlled, Non-inferiority Clinical Trial

Principal Investigator: Se-In Sung
Title: Clinical Professor

Background and Necessity of Study

I Patent ductus arteriosus (PDA) in preterm infants

1. The ductus arteriosus (DA) is a blood vessel that plays a critical role in fetal circulation. It should close at birth, but delayed closure may lead to a *persistent* PDA, heart failure, and pulmonary congestion.
2. PDA is very common among preterm infants. The incidence of PDA is higher at lower gestational age, while the incidence of hemodynamically significant PDA (hsPDA), which requires aggressive treatment, also increases.
   ① Approximately 70% of infants with birth weight < 1,000 g receive drug (ibuprofen/indomethacin) or surgical treatment.
   ② Approximately 80% of infants with gestational age ≤ 24 weeks receive surgical treatment.
3. Treatment modalities for PDA basically include conservative treatment with fluid restriction; drug treatment using ibuprofen or indomethacin; and surgical treatment, such as PDA ligation.
4. There is much controversy about treating PDA
   ① Controversy about the timing and methods of PDA treatment
      - Prophylactic administration vs. early administration vs. delayed administration; intravenous administration vs. tubal administration; indomethacin vs. ibuprofen; drug treatment vs. surgical treatment; etc.

   ② Controversy about the efficacy of PDA treatment

Delayed PDA closure → poor outcome (*TRUE*)
Treatment to induce early PDA closure → improved outcome (*TRUE???*)

      - Delayed PDA closure ⇒ poor outcome *(TRUE)*
         - The fact that delayed PDA closure in preterm infants is associated with higher mortality and morbidity of major neonatal diseases, including intraventricular hemorrhage (IVH), chronic lung disease of prematurity (CLD), and necrotizing enterocolitis (NEC) became known approximately 50 years ago (especially high association with CLD).
           → <u>Generalization of aggressive drug/surgical treatment</u>
         - A recent retrospective study also reported that delayed closure is associated with increased risk of CLD

Huston R, Wang L, Grunkemeier G, Mischel R, Cohen H, Freitag B. Permissive tolerance of the patent ductus arteriosus may increase the risk of Chronic Lung Disease. Research and Reports in Neonatology. 2013:5–10.

- Treatment to induce early PDA closure ⇒ improved outcome *(TRUE???)*
  - In a recent systematic review, meta-analysis of pooled data from existing randomized clinical trials (RCTs) showed that the drug treatment group (ibuprofen) did not show improved outcomes, as compared to the untreated group.
    - ✓ Claimed that PDA shares an association with poor outcomes, but no causality (prematurity is associated with higher PDA incidence and increased neonatal morbidity)
    - ✓ The association between PDA and poor outcomes does not provide any evidence of treatment
    - ✓ Pointed out that there are no studies that tested the efficacy on the treated group relative to the untreated group (the true control) due to strong association between PDA and poor outcomes (most studies compared the efficacy between different treatment modalities)

      Benitz WE. Treatment of persistent patent ductus arteriosus in preterm infants: time to accept the null hypothesis? Journal of perinatology: official journal of the California Perinatal Association. 2010;30(4):241–52.
- Published articles demonstrating how using only conservative treatment with no aggressive drug treatment (ibuprofen) does not worsen the mortality rate and morbidity rate for CLD, while avoiding adverse events (AEs) associated with drug treatment began to appear starting from late 2000.
  - *Vanhaesebrouck S, Zonnenberg I, Vandervoort P, Bruneel E, Van Hoestenberghe M-R, Theyskens C.* ***Conservative treatment for patent ductus arteriosus in the preterm****. Archives of disease in childhood. Fetal and neonatal edition. 2007;92(4):F244–7.*

    → Article on the efficacy of conservative treatment demonstrated by a prospective observational study; the conservative and aggressive treatment groups showed no difference in outcomes, including survival rate
  - *Noori S. Patent ductus arteriosus in the preterm infant:* ***to treat or not to treat?*** *Journal of perinatology : official journal of the California Perinatal Association. 2010;30 Suppl(S1):S31–7.*

    → Claimed that the evidence for conventional ibuprofen treatment is weak
  - *Kaempf JW, Wu YX, Kaempf a J, Kaempf a M, Wang L, Grunkemeier G.* ***What happens when the patent ductus arteriosus is treated less aggressively in very low birth weight infants?*** *Journal of perinatology : official journal of the California Perinatal Association. 2012;32(5):344–8.*

→ Article on the efficacy of conservative treatment demonstrated by a retrospective before-after observational study, outcomes equivalent to aggressive treatment were found with only conservative treatment

- *Evans N. Preterm patent ductus arteriosus:* ***should we treat it?*** *Journal of pediatrics and child health. 2012;48(9):753–8.*

  → Claimed that the evidence for conventional ibuprofen treatment is weak
- *Tauzin L, Joubert C, Noel A-C, Bouissou A, Moulies M-E. Effect of persistent patent ductus arteriosus on mortality and morbidity in very low-birth weight infants. Acta paediatrica (Oslo, Norway : 1992). 2012;101(4):419–23.*

  → Conservative treatment did not cause increased mortality or morbidity.
- *Bose CL, Laughon MM. Patent ductus arteriosus:* ***lack of evidence for common treatments.*** *Archives of disease in childhood. Fetal and neonatal edition. 2007;92(6):F498–502.*

  Benitz WE. ***Learning to live with patency of the ductus arteriosus*** *in preterm infants. Journal of Perinatology. 2011;31:S42–S48.*

  → Clarified that PDA is not something to be treated, but instead, it is an indicator that reflects the overall level of preterm infant care

③ Reasons why there were no studies that made comparisons with a true control

- Because early PDA closure was naturally pursued, drug treatment was administered repeatedly or combined with surgical treatment.
- Because the overall level of preterm infant care and conservative management for PDA were not enough to induce spontaneous ductal closure, such as fluid therapy and ventilator therapy, PDA management was impossible without drug/surgical treatment.

5 Summary (supplement)

① There were no studies that provided strong evidence of treatment, including well-designed RCTs that demonstrated the therapeutic efficacy of routinely administered ibuprofen treatment

② Articles demonstrating how using only conservative treatment with no ibuprofen treatment showed comparable mortality and morbidity (CLD) rates, while avoiding AEs associated with ibuprofen treatment, have been published in recent times.

③ Based on the lack of clear evidence demonstrating the efficacy of ibuprofen treatment and by the principle of *primum non nocere*, it was determined that conducting studies with the conservative treatment group that did not receive ibuprofen treatment as the control group would not be problematic from an ethical perspective. In other words, the researchers determined that not using ibuprofen treatment may exceed the minimal risk, but the risk-to-benefit ratio would be at an acceptable level.

④ A back-up treatment plan was presented due to concerns about the risk of not administering routine drug/surgical treatment; the study could be discontinued and the patient could receive the back-up treatment whenever necessary as requested by a guardian or determined by the medical staff

II Preliminary study

1 Based on the lack of evidence for conventional aggressive treatments for PDA

⇒ Changed the PDA treatment guidelines for preterm infants at Samsung Medical Center (SMC)

Change the PDA treatment guidelines for preterm infants (gestational age ≤ 27 weeks) admitted to the SMC neonatal intensive care unit (NICU)

① January 2010 ~ December 2011 (conventional drug/surgical treatment period)

- Drug or surgical treatment administered when cardiac workload due to PDA is determined to be present

② January 2010 ~ December 2011 (conservative treatment period)

- Administer conservative treatment regardless of PDA size and presence of cardiac workload
- Conduct a retrospective study to compare the periods

2 Preliminary study results

① Refer to Figure 1 and Tables 1 and 2 (excerpt from *Pediatric Academic Societies 2013 poster)*

② Even in extremely preterm infants with gestational age of 24 weeks, spontaneous closure was achieved with conservative treatment alone, without any drug/surgical treatment

③ Despite spontaneous and delayed closure, there was no increase of neonatal mortality rate and morbidity rate for major neonatal diseases, including IVH and NEC

In fact, protective effect for CLD was found

<Figure 1> Comparison between conventional drug/surgical treatment period (Period 1) and conservative treatment period (Period 2) groups

<Table 1> Comparison of mortality and morbidity rates by periods

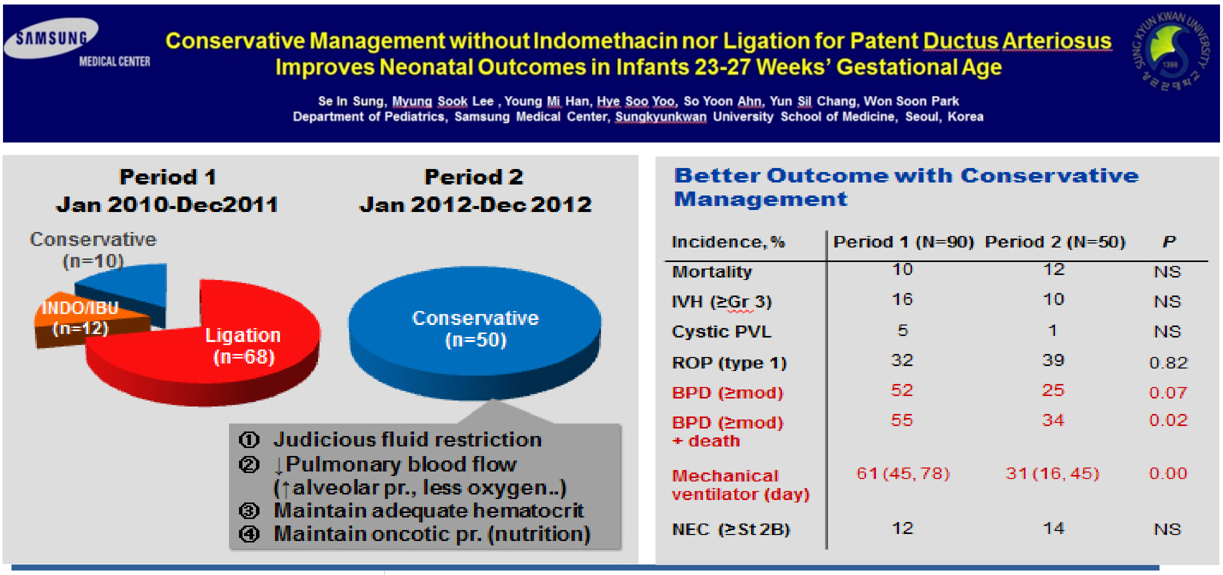

	Total (n=139)	
	Drug treatment period (n=89)	Conservative treatment period (n=50)
Demographic characteristics		
Gestational age, weeks	24.9 ± 1.3	24.7 ± 1.3
Birth weight, g	718 ± 149	712 ± 153
Antepartum steroid use, %	82	83
Pulmonary surfactant use, %	100	100
Mean PDA size, mm	2.5 ± 0.7	2.6 ± 0.8
Mortality and morbidity rates		
Mortality rate, %	10	12
IVH, %	16	10
CLD, %	52	25
CLD or death, %	55	35
NEC, %	12	14

<Table 2> Multivariate analysis (analysis of outcomes in drug/surgical treatment period versus conservative

treatment period)

	Conservative treatment period / Drug/surgical treatment period	
	Odds ratio	95% confidence intervals
Mortality rate	1.212	0.378-3.885
IVH	0.515	0.180-1.471
CLD	0.304	0.134-0.688
NEC	1.060	0.186-0.829

3 Significance of the findings in the preliminary study

① Not treating PDA caused delayed PDA closure, but the outcomes were equivalent.

② The overall level of care for extremely preterm infants improved to enable PDA management would any drug/surgical treatment

- All 141 extremely low birth weight infants born in SMC during a 1-year period (January ~ December 2012) were managed without any drug/surgical treatment ⇒ equivalent outcomes
- <u>First in the world to achieve survival of an infant with gestational age of 21 weeks</u> without any drug/surgical treatment (excluded from data above)

③ Became possible to establish a true control (group without any (drug/surgical treatment), which was impossible in existing studies

III Study Question

(1) In existing studies, early routine treatment did not show improved outcomes in preterm infants;

(2) The prospective preliminary study results showed no difference in outcomes, such as mortality rate, in the no treatment group despite delayed PDA, as compared to the conventional drug/surgical treatment group;

(3) Accordingly, the present study began with the question that wouldn't it be better not to administer conventional drug/surgical treatment, considering the AEs and complications associated with drug/surgical treatment.

(4) The objective is to prove that "outcomes in the no treatment group are not inferior to those in the conventional drug/surgical treatment group."

Objective

To prove that the outcomes in the no treatment group are not inferior to those in the ibuprofen treatment group for PDA in extremely premature infants with gestational age of ≤ 30 weeks or birth weight of ≤ 1,250 g

Necessity of study

I PDA treatment is a major issue in neonatal intensive care, but there are no established treatment

guidelines to date

(a field in which aggressive treatments have been administered for decades based on weak evidence)

II The results of this study may provide standardized domestic/international treatment guidelines

III Proof of non-inferiority could discourage unnecessary treatments, which could not only change the treatment paradigm for extremely preterm infants but also minimize the treatment-related social/economic costs.

IV Standardized treatment could lead to new clinical trials

Goal

Test the efficacy and safety of no treatment relative to ibuprofen treatment for PDA in extremely premature infants with gestational age of ≤ 30 weeks or birth weight of ≤ 1,250 g

Study details

I Subjects

1 Selection criteria (selected when all are satisfied)

① Newborns with gestational age ≤ 30 weeks and 6 days or birth weight ≤ 1,250 g

② Newborns delivered in SMC

③ Identification of hsPDA by echocardiography within 6~14 days after birth

- Definition of hsPDA
    - Respiratory assistance (high flow nasal cannula or greater) + at least one clinical criteria + ductal size ≥ 1.5 mm on echocardiography
    - Clinical criteria
        - ✓ Respiratory signs, including tachypnea, chest retraction, increased respiratory support, unable to wean respiratory support
        - ✓ Physical signs, including a murmur, hyperdynamic precordium or bounding pulses
        - ✓ Blood pressure problems, including decreased mean or diastolic pressure or increased pulse pressure
        - ✓ Signs of congestive heart failure, including cardiomegaly, hepatomegaly or pulmonary congestion

2 Exclusion criteria (excluded if at least one is satisfied)

① Mortality within 48 hours after birth

② Right-to-left shunt or bidirectional shunting with dominant right-to-left shunt through PDA

③ Comorbid with congenital deformity (congenital cardiac anomaly, etc.)

④ Bilateral IVH (grade 4 or higher)

⑤ Contraindications for ibuprofen

- Life-threatening infection
- Active bleeding, especially intracranial or gastrointestinal bleeding

- Thrombocytopenia or coagulation defect (bleeding tendency, platelet count < 10,000/mm^3)
- Significant renal dysfunction (creatinine level > 2.0 mg/dL)
- NEC (Bell's stage II or higher)

3 Dropout criteria

① Withdrawal of consent by the parent/legal representative of the subject

② Loss to follow-up due to continued no-show by the subject or the subject switching to another hospital

③ When the investigator determines that continuing to participate in the clinical trial may be harmful to the subject

④ In case of any serious AE

4 Study population: 142 patients

① Consecutive sampling

② Sample size

For the non-inferiority study, we assumed that the proportion of infants developing BPD or mortality (the primary endpoint of this study) under conservative treatment was 35%. For a one-sided test of significance with a probability of type I error (alpha) of 0.05, and assuming two equal treatment groups, the sample size required per group to achieve 80% power to detect a non-inferiority margin difference between the group proportions of 0.20 were 71 (total sample size of 142).

(1) Hypothesis: $H_0 : P_t - P_A \geq \delta$ vs $H_1 : P_t - P_A < \delta$

Where, P_T : Response rate in the treatment group;
P_A : Response rate in the control group;
δ : Tolerance of equivalence

(2) Level of significance, α=0.05 (two-sided test)
(3) With type II error (β) set to 0.2, power of the test is maintained at 80%.
(4) The ratio of subjects in the experimental and control groups is set to 1:1.
(5) Up to now, there have been no studies that found significant difference against a placebo with respect to incidence of CLD or death, which is the primary outcome in this clinical trial. Accordingly, the research team defined the limits of non-inferiority based on the preliminary study.

When CLD or mortality rate was examined after adjusting for gestational age in weeks and PDA size in the preliminary study conducted by the research team, the results showed a difference of approximately 20% between the conservative treatment period (period1; ~55%) and drug/surgical treatment period (~35%). However, this level of difference did not have an influence on the survival rate (P=0.42). Accordingly, the difference of 20% in CLD or death was established as the limit of non-inferiority (equivalence) of the two arms.

Based on the above, calculation could be made by the equation below;

$$n=\frac{(z_{\alpha}+z_{\beta})^2*2*p*(1-p)}{\{(d)-\delta\}^2}=\frac{(1.645+0.842)^2*2*0.35*(1-0.35)}{(-0.2)^2} = \text{approximately } 71$$

[α = 0.05 , β = 0.2, p = overall treatment rate, d = expected difference (except, expected difference was considered to be 0 since the hypothesis assumed that both drugs are the same), and δ = difference between the two groups]
Subjects for the experimental and control groups will be recruited at a 1:1 ratio.
Accordingly, the sample size for both groups was calculated to be 142 (due to the nature of this trial

and the subjects, dropout rate was determined to be negligible)

I Methods

1 Double-blind, randomized clinical trial

① Comparison between the treatment group (conservative treatment + ibuprofen administration) and the control group (conservative treatment + physiological saline administration)

② Randomization method

- Stratified block randomization is performed by classifying the subjects by strata according to their gestational age to maintain a balance between the treatment and control groups. Gestational age is divided into two strata (gestational age < 27 weeks and ≥ 27 weeks) and randomization is performed independently for each strata. The treatment and control groups are assigned at a 1:1 ratio to a single block and a table of randomization codes is generating using SAS program

③ Maintaining blinding

In this double-blind, randomized clinical trial, blinding is maintained until the end of the clinical trial to make sure that the investigators, subjects, and coordinator are unaware of which group each subject has been assigned to. Unblinded personnel are assigned a pharmacist or a representative and a nurse for drug administration to delegate the duties of management and administration of the investigational drug. The pharmacist or a representative and the nurse for drug administration, who are unblinded, shall be excluded from reporting of endpoints and analysis of results. Such delegation of roles shall be described and stipulated in the work delegation log prior to the start of the trial. Removal of blinding must be considered on a case-by-case basis, only in cases involving a medical emergency. Generally, blinding must be removed only when any information about the drug administration group may influence the treatment of subjects or when the principal investigator determines that removal of blinding is necessary. In such cases, records of removal of blinding must be documented and kept.

2 Administration period: With 24 hours after confirmation of PDA

3 Administration methods

① Oral administration of ibuprofen or a placebo

② Administer 10mg/kg of ibuprofen as the starting dose, followed by 5mg/kg after 24 hours and 5mg/kg after 48 hours

③ Use equivalent dose of physiological saline (mL) for the control group

4 Contraindicated concomitant drugs: Ketorolac Tromethamine, Methotrexate

The following drugs should be used with caution, considering their effects and AEs:

Diuretics (furosemide, thiazide), anticoagulants (minimal heparin for maintaining the central venous line is excluded), corticosteroids, nitric oxide, other NSAIDs, and aminoglycosides

5 Screening tests: Blood test, echoencephalography, etc.

6 Echocardiography

① PDA size, direction of blood flow, left atrium/aorta ratio, ventricular function assessment

② Assessments at screening, Day 6, Day 15, Day 29, and postmenstrual age (PMA) of 36 weeks

7 Primary endpoint

① Incidence of CLD (BPD) or death

(CLD is defined as dependency on oxygen or positive-pressure ventilation at correct age of 36 weeks; death is defined as death up to corrected age of 36 weeks)

8 Secondary endpoints

① PDA closure rate (at day#7 after randomization, at CA 36 weeks, until hospital discharge)

② Mortality rate up to corrected age of 36 weeks and 40 weeks, morbidity rate for CLD (CLD is defined as dependency on oxygen or positive-pressure ventilation at correct age of 36 weeks)

③ Respiratory status at corrected age of 40 weeks

④ Prevalence of major neonatal diseases

- Unilateral or bilateral IVH (grade 3 or higher)
- Retinopathy of prematurity (ROP; stage 3 or higher)
- NEC (stage 2b or higher)
- Sepsis, as determined by positive blood culture test result

⑤ Long-term neurodevelopmental outcomes – Presence of cerebral palsy, bilateral hearing and/or visual impairment during the trial period, Bayley Scales of Infant Development (BSID-II) at corrected age of 18-24 months, growth parameters, etc.

9 Other variables

Gestational age, birth weight, sex, small-for-gestational age, method of delivery, history of antepartum steroid use, chorioamnionitis, gestational hypertension, Apgar score, pulmonary surfactant use, myocardial constrictor use, dexamethasone use, echocardiography results (PDA size, left atrium/aorta ratio), blood test results (kidney function values, etc.), time point of full enteral feeding, sepsis, intestinal surgery, presence and time of PDA occlusion, duration of dependency on mechanical ventilator (duration of intubation), etc.

10 Safety assessment and reporting methods, including AEs

9.1 Definition of AE

AE refers to an unfavorable and unintended sign, symptom, and disease that occur in a subject during the clinical trial, which does not necessarily have a causal relationship with the investigational drug. Adverse drug reaction (ADR) refers to a harmful and unintended reaction which occurs at an arbitrary dose of the investigational drug, in which, a causal relationship with the investigational drug cannot be entirely dismissed.

Serious AE/ADR refers to one of the following among AEs or ADRs that occurred at an arbitrary dose of the investigational drug;

① Causing death

② Life-threatening

③ Causing hospitalization or prolonging the length of hospital stay

④ Causing persistent or serious disability or impairment

⑤ Causing congenital malformation

- Considering that the subjects are extremely preterm infants, the following SAEs are anticipated
  - ■ Death, IVH (grade 3 or higher), NEC (Bell's stage 2b or higher), ROP (grade 4 or higher), pulmonary hemorrhage, and oliguric acute renal failure with serum creatinine level ≥ 3.0mg/dL

9.2 Assessment of AEs

The investigators shall assess the severity of AEs by the following criteria.

Grade	Criterion
Grade 1	Mild AE
Grade 2	Moderate AE
Grade 3	Severe AE
Grade 4	Life-threatening or disabling AE
Grade 5	Fatal AE

The investigators shall assess the causal relationship between AEs and the investigational drug by the following criteria.

① Definitely related

② Probably related

③ Possibly related

④ Not related

⑤ Unknown

9.3 Reporting of AEs

The investigators shall collect information about all AEs and SAEs that occur within 24 hours from the administration of the starting dose to the final dose; report them according to relevant regulations; and keep records of such events in the case report form (CRF).

Until the completion of the clinical trial, the investigators shall report any suspected unexpected serious adverse drug reaction (SUSAR) to the Institutional Review Board (IRB) and the Ministry of Food and Drug Safety (MFDS) in timely manner.

The investigators must report any SUSAR within the following timeframe.

① SUSAR causing death or life-threatening: Within 7 days of the investigator becoming aware or receiving a report of such SUSAR (additional reporting of details within 8 days from the first report)

② Other SUSARs: Within 15 days of the investigator becoming aware or receiving a report of such SUSAR

11 Statistics

① Use of Stata 13

- ▪ Per protocol
  - ▫ Subjects who completed the study per protocol up to the final visit among those

included in the FAS group. Protocol violation is defined by the following criteria.

(1) Subjects who did not satisfy both the selection and exclusion criteria

(2) Subjects with medication adherence < 70%

(3) Subjects who received any drug therapy or other treatment unauthorized by the physician in charge during the study period, which may influence the determination of the clinical trial results

(4) Other serious protocol violations that may influence the determination of the clinical trial results

- ▫

- ▪ Primary endpoint: Comparison of differences between the two groups by χ^2 test
  - ▫ A non-inferiority test will be performed for comparison between the experimental and control groups. After estimating the 95% confidence interval for the difference in incidence of CLD and mortality between the experimental and control groups, if the lower limit of the estimated confidence interval is greater than the pre-determined non-inferiority margin of -20%, then the effect in the experimental group is determined to be not inferior to that of the control group.
- ▪ Secondary endpoints:
  - ▫ Mortality rate and morbidity rate for CLD (CLD is defined as dependency on oxygen or positive-pressure ventilation at correct age of 36 weeks) up to 40 weeks, respiratory status (oxygen or ventilator dependency) at correct age of 40 weeks, unilateral or bilateral IVH (grade 3 or higher), ROP (stage 3 or higher), NEC (stage 2b or higher), and sepsis determined by positive blood culture test result are compared and analyzed. Discreet variables are compared by χ^2 test and continuous variables are compared and analyzed by independent T-test or Mann-Whitney U test.
  - ▫ Analysis of long-term neurodevelopmental outcomes: Discreet variables are compared by χ^2 test and continuous variables are analyzed by independent T-test or Mann-Whitney U test

② Subgroup analysis by gestational age

- ▪ After dividing both groups into subgroups (group1: gestational age < 27 weeks and group 2: gestational age ≥ 27 weeks), the subgroups are compared by χ^2 test.

③ Analysis time points

- ▪ Primary and secondary endpoints, excluding long-term neurodevelopmental outcomes, are completed by corrected age of 40 weeks (Visit 8), and if assessment of all subjects is completed by that time, preliminary analysis will be performed on the relevant endpoints. At this time, the unblinded personnel will secretly deliver the randomization code table to the statistics team, and as a result, blinding of the blinded part researchers, excluding the statistics team, will be maintained. Blinding is also maintained in the subsequent analysis of long-term neurodevelopmental outcomes during Visit 9.

- Secondary analysis of long-term neurodevelopmental outcomes
  - Evaluation of long-term neurodevelopmental outcomes will take place after the preliminary analysis, which could raise concerns about removal of blinding. However, because the unblinded part will secretly deliver the randomization table to the statistics team, blinding of the blinded part researchers will be maintained and would not influence the long-term neurodevelopmental outcomes. Moreover, evaluation of long-term neurodevelopmental outcomes will be performed by ophthalmologists, otolaryngologists, and occupational therapists belonging to the division of rehabilitation medicine, who are not affiliated with the researchers belonging to the division of pediatrics. It is determined that blinding will be maintained since there will be no exchanges or conflict of interest with them.

12 Clinical trial procedures and management regulations

① Confidentiality

- By signing the approval form, the principal investigator acknowledges that all information related to the clinical trial will be kept confidential. In addition, the principal investigator also acknowledges that when information is disclosed to the IRB or ethics committee, such information shall be provided after assuring sufficient understanding of the confidential nature of the information.
- Randomly assigned subject identification codes will be used to conceal personal information.

② Potential risks, benefits, alternative treatments, and indemnification

- AEs associated with ibuprofen treatment include transient oliguria and renal dysfunction, while ibuprofen has been reported to increase the risk of NEC in newborns. The aforementioned AEs are commonly found when treating preterm infants, regardless of ibuprofen treatment, and treatment for AEs are already well known. The researchers will attempt to do their best to detect such AEs early and treated as best they can.
- Risks associated with not administering ibuprofen are at acceptable level, which could mostly be addressed with medical treatment. However, if the patient's condition worsens and persistent PDA is suspected to be the cause, then ibuprofen or surgical treatment may be considered as a back-up treatment.
  - Criteria of back-up treatment

    (1) persistent PDA persists, (2) which causes persistent heart failure symptoms (hypotension, elevated pulse pressure, oliguria, cardiomegaly, feeding difficulties, and increased dependency on mechanical ventilation), (3) and such symptoms are not caused by prematurity itself or other factors, such as infection.
- There are no direct benefits for the subjects from participating in this clinical trial, but the findings in the study are expected to have social benefits by providing evidentiary data for establishing treatment guidelines for preterm infants who have the same disease.
- Other treatment options besides ibuprofen therapy and conservative treatment include

indomethacin therapy and surgical treatment. However, ibuprofen is known to have fewer AEs than indomethacin, while early surgical treatment may be harmful (Cochrane database of systematic review).

- Indemnification for injuries related to the clinical trial

  If any subject registered in this clinical trial requires treatment for an injury caused directly by the investigational drug (ibuprofen or physiological saline) during the study period, the principal investigator will indemnify the subject for such injury through the clinical trial insurance. If any subject incurs bodily injury from participating in this clinical trial, appropriate treatment or treatment opportunity will be provided first by the principal investigator even if monetary compensation for such injury has not be settled.

Indemnification policy

- If any subject incurs bodily injury from participating in this clinical trial, the subject shall be compensated accordingly.
- A) Bodily injury to the subject (including death)
- B) Injury incurred due to administration of investigational drug or during corrective treatment of an AE
- C) Among injuries incurred in relation to the clinical trial, those for which the investigators cannot prove no causal relationship with the investigational drug

Treatment of AEs must meet the following conditions.

- During their participation in the clinical trial, the subjects must strictly adhere to the study protocol and instructions given by the principal investigator and co-investigators.
- All AEs must be reported immediately to the principal investigator and co-investigators for preparation of appropriate actions.

Indemnification does not cover the following:

- Injuries attributable to accidents that could occur even during times besides the clinical trial
- Compensation for not meeting the expected effect and/or efficacy of the investigational drug
- All costs related to injuries caused by participation in activities beyond the scope of this study protocol or personal actions by the subject or guardian (including injuries from tests or treatments not required in the study protocol)
- Injuries incurred due to intentional or gross negligence and/or non-compliance with the study protocol and instructions given by the investigators by the subject and/or guardian
- Injuries due to typical complications that may occur as a result of exacerbation or natural progression of any underlying disease
- Monetary compensation claimed due to loss of income, disability, and/or discomfort or

loss claimed by a spouse or family member, including community loss

<Table 3> Study schedule chart

Study day	Screening	D#0	D#3	D#6	D#15	D#29	PMA 36wk	PMA 40wk	Corrected age 18-24mo
Visit no.	Visit 1	Visit 2	Visit 3	Visit 4	Visit 5	Visit 6	Visit 7	Visit 8	Visit 9
Visit window				± 1 day	± 2 days	± 2 days	± 1 week	± 2 weeks	
Informed consent	**X**								
Randomization		**X**							
Ibuprofen or placebo (physiological saline) administration		**X**3)							
Vital signs	**X**1)	**X**	**X**	**X**	**X**	**X**	**X**	**X**	
Physical examination/ Anthropometric measurements (weight, height, and head circumference)	**X**1)	**X**2)			**X**	**X**	**X**		
CBC/Chemistry	**X**1)				**X**				
NT-proBNP	**X**1)				**X**				
Chest X-ray	**X**1)	**X**	**X**	**X**	**X**				
Echocardiogram	**X**1)			**X**6)	**X**6)	**X**6)	**X**6)		
Head US	**X**1)								
AE collection5)		**X**	**X**	**X**	**X**	**X**	**X**	**X**	**X**
Concomitant medication4)	**X**	**X**	**X**	**X**	**X**	**X**	**X**	**X**	
Endpoint data collection							**X**	**X**	
Neurodevelopmental evaluation									**X**

- Screening and investigational drug administration may occur on the same day (D#0) (assessment of screening results must be completed before random assignment)

1) **:** This could be used if data from within 5 days of the screening (within 7 days for head US) are available. Except, data from within 24 hours could be used for echocardiography. Vital signs could be omitted as determined by the investigator.

2) : Only weight data (D#0) are collected; weight data are used to determine the dose of investigational drug.

3) : Ibuprofen or placebo (physiological saline) is administered within 24 hour after confirmation of PDA with a starting dose of 10mg/kg per os (PO), followed by 5 mg/kg PO after 24 hours and 5mg/kg PO QD after 48 hours at the same time, if possible.

4) : With respect to concomitant medication, information about only dexamethasone, cardiotonic agents (dopamine and dobutamine), and diuretics (furosemide) is collected. Information collected shall included the name, dose, duration, and purpose of use of dexamethasone, cardiotonic agents, and diuretics used from birth to discharge.

5) : The investigators shall collect information about AEs and SAEs that occur within 24 hours from the administration of the starting dose to the final dose; report them according to relevant regulations; and keep records of such events in the CRF. Until the completion of the clinical trial, the investigators shall report all SUSAR to the IRB and MFDS in timely manner.

6) : Upon confirmation of 2 consecutive PDA closure after administration of the investigational drug, tests may be omitted as determined by the investigator.

Utilization plan and spin-off effects after completion

I Utilization plan

1 Evidence for establishment of domestic/international PDA treatment guidelines for preterm infants

- Evidence-based treatment guidelines

II Spin-off effects

1 Establish evidence-based guidelines for treating PDA in preterm infants, which had been performed for decades without any standardized treatment guidelines

2 Depending on the findings in the study, a) if combination of conservative treatment + drug treatment is superior: serves as evidence for such treatment; b) if conservative treatment alone is not inferior: unnecessary treatment could be discouraged

① Drug treatment-related AEs and complications are maximized in preterm infants

② Risks and complications associated with surgical treatment by an open chest operation are very serious in preterm infants

③ Treatment cost savings

3 Paradigm shift in the treatment of PDA in preterm infants

① Standardized treatment could lead to new clinical studies

② Present know-how for conservative treatment of PDA in clinical practice (Learning to live with PDA)

III Personal information protection and data confidentiality plan

All information pertaining to this clinical trial shall not be disclosed or provided to any outside party and shall not be used for any purposes other than the study objectives. In addition, all investigators, managers, and staff participating in the study shall strictly adhere to the confidentiality clause. All personally identifiable information and records shall be kept confidential and such personally identifiable information shall not be included in any form in articles or reports even if the findings of this clinical trial are published.

Sample size calculation

1. Sample size

   A. For the non-inferiority study, we assumed that the proportion of infants developing 'BPD or death' (the primary endpoint of this study) under conservative treatment was 35%. For a one-sided test of significance with a probability of type I error (alpha) of 0.05, and assuming two equal treatment groups, the sample size required per group to achieve 80% power to detect a non-inferiority margin difference between the group proportions of 0.20 were 71 (total sample size of 142).

   B. Hypothesis: $H_0 : P_t - P_A \geq \delta$ vs $H_1 : P_t - P_A < \delta$

   Where, P_T: response rate in the treatment group

   P_A: response rate in the control group

   δ: non-inferiority margin

   Level of significance, α=0.05 (two-sided test)

   With type II error (β) set to 0.2, power of the test is maintained at 80%.

   The ratio of subjects in the experimental and control groups is set to 1:1.

2. Limit of non-inferiority

   A. ICH E10 guideline states that the determination of the margin in a non-inferiority trial should be based on both statistical reasoning and clinical judgement, and should reflect uncertainties in the evidence on which the choice is based, and should be suitably conservative.

      B. Statistical reasoning: The noninferiority margin of 20% was defined on the basis of the lower limit of the 95% confidence intervals (19.8 to 20.2) of the difference in BPD incidence or mortality between the nonintervention group (18/51, 35%) and treatment group (49/73, 55%) in our historical data.

      C. Clinical judgement: As developing BPD/death could be affected not only by PDA management but also by diverse and complex host factors and variations in clinical practice, despite the risk of falsely justifying the efficacy of nonintervention over pharmacologic treatment with relatively large noninferiority margin, we assumed that 20% of noninferiority margin would be clinically acceptable. Therefore, on the basis of statistical reasoning and clinical judgement, we set the margin of non-inferiority at 20% in this study.

3. Sample size calculation

   A. Based on the above, calculation could be made by the equation below;

$$N = \frac{(z_{\alpha}+z_{\beta})^2 * 2 * p * (1-p)}{\{(d)-\delta\}^2} = \frac{(1.645+0.842)^2 * 2 * 0.35 * (1-0.35)}{(-0.2)^2} = 71$$

[α = 0.05 , β = 0.2, p = overall treatment rate, d = expected difference (except, expected difference was considered to be 0 since the hypothesis assumed that both drugs are the same), and δ= Non-inferiority margin]

B. Accordingly, the sample size for both groups was calculated to be 142.
