# Supplementary material for: Effect of Nonintervention vs Oral Ibuprofen in Patent Ductus Arteriosus in Preterm Infants: A Randomized Clinical Trial
Source: JAMA Pediatr. 2020 Jun 15;174(8):1–9. doi: 10.1001/jamapediatrics.2020.1447 (PMC7296457; doi:10.1001/jamapediatrics.2020.1447)
Supplement: Supplement 2. — Data Sharing Statement [file jamapediatr-174-755-s002.pdf]

Data Sharing Statement

Sung. Effect of Nonintervention vs Oral Ibuprofen in Patent Ductus Arteriosus in Preterm Infants. *JAMA Pediatr*. Published June 15, 2020. 10.1001/jamapediatrics.2020.1447

Data
Data available: Yes
Data types: Deidentified participant data
How to access data: wonspark@skku.edu
When available: With publication

Supporting Documents
Document types: None

Additional Information
Who can access the data: Researchers whose proposed use of the data has been approved
Types of analyses: For any purpose
Mechanisms of data availability: After approval of a proposal
